# Asymptomatic seminal infection of herpes simplex virus: impact on male infertility

**DOI:** 10.7555/JBR.27.20110139

**Published:** 2012-04-20

**Authors:** Seyed Hamidreza Monavari, Mostafa Salehi Vaziri, Mohammadali Khalili, Mahmoud Shamsi-Shahrabadi, Hossein Keyvani, Hamidreza Mollaei, Mehdi Fazlalipour

**Affiliations:** a Department of Virology and Anti-Microbial Resistant Research Center, Tehran University of Medical Sciences, Tehran 1417613151, Iran;; b Department of Virology, Tehran University of Medical Sciences, Tehran 1417613151, Iran;; c Research and Clinical Center of Infertility, Yazd 8916877391, Iran.

**Keywords:** infertility, herpes simplex virus, real-time PCR, semen

## Abstract

In more than half of infertile men, the cause of their infertility is unknown. Several studies revealed the role of viral infections in male infertility. The aim of the present study was to determine the prevalence of herpes simplex virus-1 (HSV-1) and HSV-2 in semen from asymptomatic infertile male patients, and its association with altered semen parameters. A total of 70 semen samples were collected from infertile men who attended the Research and Clinical Center for Infertility in Yazd, Iran. Semen analysis and diagnostic real-time PCR using specific primers and probes for HSV-1 and HSV-2 DNA were performed. Comparison of semen parameters between virally infected and non-infected samples were performed with independent *t*-test and Mann-Whitney test. Semen analysis showed that infertile men fell into two groups, the male factor group and the unexplained group. HSV-1 and HSV-2 DNA was detected in 16 (22.9%) and 10 (14.3%) of 70 semen samples, respectively. All HSV-positive samples had abnormal semen parameters (the male factor group). Although HSV infection was not associated with sperm motility and morphological defects, it was correlated with lower sperm count in the seminal fluid. The findings suggest that asymptomatic seminal infection of HSV plays an important role in male infertility by adversely affecting sperm count.

## INTRODUCTION

Infertility, a major problem of modern medicine, is defined as the inability of sexually active couples to achieve pregnancy after 12 months. Infertility affects nearly 20% of reproductive-aged couples, in which 40%-50% of these cases are male infertility[1],[2]. The major causes of male infertility include varicocele, endocrine disturbances, immunological conditions, genital duct obstruction, gonadotoxins, medications, cryptorchidism, infection, sexual dysfunction, and ejaculatory failure[3]. About 70% of cases with male infertility are idiopathic (of unknown etiology)[3],[4]. Several microorganisms, such as chlamydia, gonococcus, mycoplasma and herpes simplex viruses (HSV) can colonize in the male genital tract[5].

Seminal tract infections may play a contributing role in male infertility. Infections impair fertility by different mechanisms, including damaging spermatogenesis, impairment of sperm function, and obstruction of the seminal tract[6],[7]. There is increasing evidence that viral infections play a role in the pathogenesis of male infertility[8]–[10]. Viral infections impair male fertility, either by directly invading the male genital tract cells, or by indirectly causing local inflammatory or immunological responses that could deteriorate reproductive functions[11]. In addition, pro-inflammatory cytokines and reactive oxygen species (ROS) may play an important role in infertility[12]. ROS may damage fertility by decreasing polyunsaturated fatty acid(PUFA) on sperm membrane, including DNA damage and impairing acrosomal reaction[12],[13].

HSV is one of the most common viruses in human populations, and is responsible for a broad spectrum of diseases, including gingivostomatitis, keratoconjunctivitis, encephalitis, neonatal infections, and genital diseases[14],[15]. HSV includes two distinct, but closely related viruses, namely, HSV-1 and HSV-2. Both viruses can cause genital herpes. Genital herpes can be asymptomatic at the time of primary, initial, or recurrent infection[16],[17]. The association between HSV infections and male infertility has been investigated, and some studies reported the association of HSV infection with infertility[18]–[20].

The aim of this study was to determine the prevalence of *HSV-1* and *HSV-2* using real-time PCR in the semen of a randomized asymptomatic infertile male group attending an infertility clinic in Iran. In addition, the possibility of HSV infections affecting semen parameters, and thus fertility, has been assessed.

## MATERIALS AND METHODS

### Samples

A cross-sectional study was designed for detection of HSV-1 and HSV-2 DNA in the semen of infertile men. Semen samples were collected from 70 men who attended the Research and Clinical Center for Infertility in Yazd, Iran. Informed consent was obtained from all participants following a detailed description of the purpose of the current study. None of the men or their spouses had reported any clinically confirmed genital herpetic infection in their medical history. In all cases, a complete semen analysis, including sperm count, motility, and morphology was performed.

### Semen analysis

Semen analysis was performed according to the WHO 2010 criteria[21]. The sperm count and motility were determined using a Meckler counting chamber under a phase contrast microscope (Nikon, Japan). The sperm was fixed in methanol, dried and stained with Giemsa, and then the morphology was evaluated by using a light microscope (Nikon, Japan).

### DNA extraction from semen samples

After collection of the specimens, each semen sample was centrifuged at 770 *g* for 10 minutes. The supernatant was removed and the pellet was transferred to an Eppendorf tube. DNA extraction was performed using the High Pure PCR Template Preparation kit (Roche Diagnostic GmbH, Mannheim, Germany) following the manufacturer's protocol. All DNA samples were subjected to spectrophotometry for quantification of DNA at 260 nm.

### Real-time PCR

All samples were tested for the presence of HSV-1 and HSV-2 DNA by real-time PCR method (Rotor Gene 6000, Corbett Research, Australia). Primers were synthesized against the highly conserved glycoprotein D gene of HSV-1 and HSV-2 to detect both viruses, and their sequences were as follows: 5′-CGCATCAAGACCACCTCCTC-3′ (sense); 5′-GCTCGCACCACGCGA-3′ (antisense). Two specific probes were synthesized against different regions in HSV-1 and HSV-2 glycoprotein D to differentiate between them, and their sequences were as follows: *HIV-1*: FAM-TGGCAACGCGGCCCAAC-BHQ1;*HIV-2*: JOE-CGGCGATGCGCCCCAG-BHQ1, which were designed using Primer3 plus online tools (www.bioinformatics.nl/cgi-bin/primer3plus/primer3plus.cgi). Briefly, 10 µL DNA was added to 10 µL reaction mixture containing 2 U *Taq*Man polymerase, 0.01% gelatin, 0.6 µmol/L each primer, 0.2 µmol/L probe, 200 µmol/L each deoxynucleotid triphosphate, 5 µL reaction buffer (50 mmol/L KCl, 10 mmol/L Tris-HCL, pH = 8.3) and 3 mmol/L MgCl_2_. PCR was performed at 95°C for 10 minutes, followed by 45 cycles of 95°C for 15 seconds, 60°C for 40 seconds and fluorescence detection in fluorometer channel FAM (Green), for HSV-1 at 60°C. Before the runs were started, the tubes were kept at 50°C for 2 minutes for activation of UNG. Standard precautions were taken to avoid sample-to-sample contamination and PCR product carryover. DNA extracted from the HSV-1 KOS strain, HSV-2 G strain and infected Vero cells served as positive controls, while a mixture without DNA template was used for negative controls.

### Statistical analysis

All statistical analyses were performed using the statistical software SPSS version 13.0 (SPSS Inc., Chicago, IL, USA). Comparison of the mean sperm count, motility and morphology between virally infected and non-infected samples was performed with the independent *t*-test and Mann-Whitney test. A *P*-value < 0.05 was considered as statistically significant.

## RESULTS

HSV-1 DNA was detected in 16 (22.9%) and HSV-2 DNA in 10 (14.3%) of 70 semen samples by real-time PCR, respectively (***Table 1***). Interestingly, all HSV-2 DNA positive samples were also positive for HSV-1 DNA, suggesting co-infection of HSV-1 and HSV-2 in HSV-2-infected subjects. Semen analysis of positive and negative samples for HSV-1 and HSV-2 DNA revealed that all positive samples had abnormal semen parameters (male factor group). The criteria for categorization of male factor and unexplained groups were shown in ***Table 2***. Cases which have each of male factor group parameters classifed in this group, but cases who exhibit all unexplained group parameters must be categorized in unexplained groups. In HSV-1 positive samples, the sperm count was (27.6±16.7)×10^6^/mL, which was significantly lower than that in those who tested-negative for HSV-1 and HSV-2 [(60.3±48.2)×10^6^/mL] (***Table 3***). In those who were positive for HSV-2, the sperm count was (39.3±7.9)×10^6^/mL, and in those who were negative for HSV-2, the sperm count was (55.1±48.3)×10^6^/mL(*P* > 0/05, ***Table 4***). Statistical analysis by independent t-test revealed no significant correlation between HSV-2 infections and abnormal sperm parameters. However, a significant relationship between HSV-1 infection and reduced in sperm count, but not with sperm motility and morphology, was detected.

**Table 1:** Positive and negative for HSV DNA samples by real time PCR (n=70)

Virus type	Positive (%)	Negative (%)
HSV-1 DNA	16 (22.9)	54(77.1)
HSV-2 DNA	10(14.3)	60(85.7)

**Table 2:** Categorization parameters of male factor and unexplained groups based on the WHO criteria (n=70)

Sperm parameters	Male factor group	Unexplained group
Sperm count (10^6^/mL)	< 20	≥20
Quick progressive motility(%)	< 50	≥50
Slow progressive motility(%)		
Normal morphology (%)	< 30	≥30

**Table 3:** Semen parameters in HSV-1 DNA positive and negative groups

Sperm parameters	HSV-1	Mean±SD	*P*
Count (×10^6^/mL)	Positive	0027.6±16.7	< 0.001
	Negative	0060.3±48.2	
Quick progressive motility(%)	Positive	003.5±5.4	0.776
	Negative	004.0±7.1	
Slow progressive motility(%)	Positive	0026.2±11.5	0.482
	Negative	0023.4±14.5	
Non-progressive motility(%)	Positive	016.1±3.2	0.418
	Negative	016.1±7.9	
Normal morphology(%)	Positive	018.6±8.6	0.423
	Negative	016.5±9.7	

**Table 4:** Semen parameters in HSV-2 DNA positive and negative groups

Sperm parameters	HSV-2	Mean±SD	*P*
Count (×10^6^/mL)	Positive	39.3±7.9	0.801
	Negative	055.1±48.3	
Quick progressive motility (%)	Positive	05.4±6.2	0.065
	Negative	03.6±6.7	
Slow progressive motility (%)	Positive	030.7±12.6	0.104
	Negative	022.9±13.8	
Non-progressive motility (%)	Positive	16.8±3.2	0.571
	Negative	15.9±7.6	
Normal morphology (%)	Positive	22.2±8.0	0.061
	Negative	16.1±9.7	

## DISCUSSION

About 50% of infertility cases are due to male factor. In the majority of male infertility cases, the cause of infertility remains unknown[22]. The role of viral infections in male infertility has been investigated, and many previous studies revealed that HSV infections were related with abnormal sperm parameters[8]–[10]. This study was designed to investigate the prevalence of HSV-1 and HSV-2 DNA in the semen of infertile men using real-time PCR, a sensitive technique for detection of infectious agents. In addition, the association between the presence of HSV-1 and HSV-2 DNA and semen parameters was investigated.

The results showed that 16 (22.9%) and 10 (14.3%) of the 70 semen samples were positive for HSV-1 and HSV-2 DNA, respectively. el Borai et al.[20] revealed a significant association between HSV and infertility. They detected HSV-1 DNA in 24% of semen samples from infertile men using a nested PCR technique. In another study by Kapranos et al.[23], HSV DNA was detected in 49.5% of semen samples and HSV infection was significantly related to low sperm count as well as poor motility. Klimova and colleagues[24] observed that seminal HSV infection was more frequently present in male infertile patients than controls, and they revealed that HSV infection was directly correlated with the reduced amount of actively motile sperm. In addition, Abdulmedzhidova et al.[25] reported that HSV was detected in 25% of males with infertility and HSV infection was associated with oligospermia and sperm structural abnormality. Kotronias et al.[26] detected HSV-1 and HSV-2 infections in the semen of 21% and 20% of infertile men, respectively. Moreover, HSV infection was related with reduced sperm count and progressive motility. Consistent with these studies, a significant relationship between HSV-1 infection and low sperm count was observed in the current study. Neofytou et al.[27] detected 2.1% of HSV-1 DNA and no HSV-2 DNA in the semen samples of infertile men. However, there was no association between the presence of HSV-1 DNA and abnormal semen parameters. In a study to determine the prevalence of pathogens that caused sexually transmitted infections in semen from asymptomatic male infertility patients, 3.7% of cases harbored HSV DNA, and among all the pathogens studied, the most robust adverse effect on both quality and levels of accessory gland markers was associated with HSV[28]. Another study by Wu et al.[29] indicated the association of HSV infection with hampered spermatogenesis, increased apoptotic cells and low sperm concentration. Moreover, some exploratory investigations on transgenic mice also exhibited evidence in favor of association between HSV infection and male infertility.

Expression of HSV thymidine kinase (HSV-tk) in transgenic mouse testis is correlated with sperm structural abnormalities, defects in spermatogenesis and increase in number of apoptotic germ cells[30]–[32]. Moreover, the decrease of HSV-tk levels leads to significant reduction of sperm abnormalities and fertility rehabilitation in mice[30],[33]. Interestingly, treatment of HSV positive male infertile patients with anti-viral drugs leads to several healthy pregnancies[20],[26]. Significant importance of HSV infections is observed in *in-vitro* fertilization techniques, and the infections are responsible for the high rate of failed fertilization[34],[35]. Importantly, HSV may cause asymptomatic persistent infection in the semen with a very low copy number yielding negative cultures[16],[36],[37]. Therefore, use of molecular techniques such as PCR, increases the possibility of detection of HSV in such cases.

In conclusion, the present study is the first to investigate the correlation between HSV infection and infertility among Iranian men, and indicates that HSV, by affecting the most important semen parameter sperm count, plays an important role in male infertility. Treatment with approved anti-HSV drugs such as acyclovir, controls HSV lytic infection. Therefore, early detection of this virus using sensitive and specific methods like PCR enables us to reduce the abnormal semen parameters and the possibility of infertility as well as to control the transmission HSV infection. Using real-time PCR assay, we detected a considerable prevalence of HSV DNA in semen from asymptomatic infertile males. HSV can be easily transmitted to the partner and cause genital lesions in mothers as well as severe problems such as encephalitis in newborns. Thus, early diagnosis and appropriate anti-viral therapy of asymptomatic genital HSV infection should be purpused.
